# Enhanced fetal MRI diagnosis of esophageal atresia using super-resolution slice-to-volume reconstruction

**DOI:** 10.1007/s00247-025-06309-z

**Published:** 2025-07-19

**Authors:** Delaney Loken, Luis F. Goncalves, Mittun Patel, Nicholas Rubert

**Affiliations:** 1 Creighton University, 3100 N Central Ave, Phoenix, AZ 85012 United States; 2 Phoenix Children’s Hospital, Phoenix, AZ United States; 3 Mayo Clinic, Scottsdale, United States; 4 University of Arizona College of Medicine, Phoenix, United States

**Keywords:** Esophageal atresia, Fetal MRI, Slice-to-volume reconstruction

## Abstract

**Background:**

Prenatal diagnosis of esophageal atresia remains challenging, with indirect signs such as polyhydramnios, a small or absent stomach bubble, and a dilated upper esophageal pouch often inconsistently present. Only 10-40% of EA cases are diagnosed prenatally. Fetal MRI can overcome ultrasound limitations; however, constraints like motion can hinder evaluation of the esophagus.

**Methods:**

Super-resolution imaging with slice-to-volume reconstruction (SVR) is one approach that can improve image quality. This technique generates high-resolution 3D images from standard fetal MRI slices to enhance diagnostic accuracy.

**Aim:**

We present the application of super-resolution imaging with SVR to accurately diagnose EA and assess the presence or absence tracheoesophageal fistulas.

**Conclusion:**

This technique demonstrates significant potential for accurately delineating the relevant surgical anatomy, which can improve surgical planning.

## Introduction

Esophageal atresia is a developmental defect in which the upper and lower esophagi fail to connect and can occur with or without the presence of a tracheoesophageal fistula [1]. Prenatal diagnosis allows healthcare providers to evaluate the severity of the malformation, identification of associated abnormalities, parental counseling, surgical planning, and decreased transportation time to a surgical unit [1, 2]. Despite the importance, prenatal diagnosis is often difficult because indirect signs like polyhydramnios (presents in 60% of fetuses with esophageal atresia), small or absent stomach bubbles (presents in 50%), and dilated upper esophageal pouch are nonspecific [2, 3]. Polyhydramnios, the most common indirect sign of esophageal atresia, is present in about 10% of pregnancies and can be associated with many different congenital anomalies involving impaired fetal swallowing [3]. The overall reported sensitivity of ultrasound to diagnose esophageal atresia is approximately 41.9% [3].

Fetal MRI has been used for prenatal diagnosis since the early 1990 s [4]. A meta-analysis published in 2019 [3] suggested that fetal MRI may have a high diagnostic sensitivity (88.9%; 95% CI, 80.2–95.8%) and specificity (99.6%; 95% CI, 98.7–100%) for the diagnosis of esophageal atresia. These data, however, should be interpreted with caution due to the small number of included studies (*n* = 5) and fetuses (*n* = 99), as well as the potential for spectrum bias—MRI was typically used as a secondary imaging modality in cases with high clinical suspicion, potentially overestimating diagnostic performance [3]. There are limitations to using fetal MRI as it relies on the same indirect signs as ultrasound. Typically, fetal MRI image quality is determined based on both signal-to-noise ratio (SNR) and the presence or absence of motion [5]. The lower the SNR and the higher the amount of movement of the fetus, the lower the image quality. On modern hardware, 2D single-shot fast spin echo (SS-FSE) images have relatively high SNR. However, 2D-SS-FSE image resolution is anisotropic, and the 3D appearance of the images may be corrupted by motion [5]. One way to help overcome this is to use super-resolution imaging with slice-to-volume reconstruction (SVR) [5]. SVR corrects for motion in fetal MRI and can be exceptionally useful in parts of the body where motion can degrade the quality of the image. SVR gives us the ability to create high-resolution 3D images from multiple 2D slices which improve anatomical visualization. To create the 3D SVR images, nine sets of 2D-SS-FSE images with different orientations for the slice-select axis were acquired (FOV 224 × 224 mm, slice thickness 3 mm with no gap, 1.5-T Philips Achieva dStream, Philips Healthcare, Amsterdam, The Netherlands) and transferred from Philips Intellispace PACS 4.7.17 (Philips Healthcare, Amsterdam, The Netherlands) to a research server. On the research server, images in DICOM format were converted from DICOM format to NIfTI format using NiBabel 3.2.2 (open-source software maintained by the NiPy community, available at https://nipy.org/nibabel). A mask image of the area to be reconstructed was then created from a single 2D acquisition using ITK-SNAP 4.0 (Penn Image Computing and Science Laboratory, University of Pennsylvania, Philadelphia, PA, USA). The super-resolution 3D volume was reconstructed from all nine stacks using the deformable SVR package of SVRTK (git commit ID: 6bbe1a76881ac2aaa1baed132be89cf5bdcbb92b), as described by Uus et al. [5]. The output of SVRTK was converted into a DICOM format and transferred into Philips Intellispace Portal software for visualization and 3D rendering. Transfer of images to and from the research server and launching of Python scripts to handle file conversion and reconstruction were managed with Agora 7.6.1 (Agora, Houston, TX, USA). We applied this technique to cases with suspected esophageal atresia to see if it would provide more information compared to the original images.

## Case

**Fig. 1:**
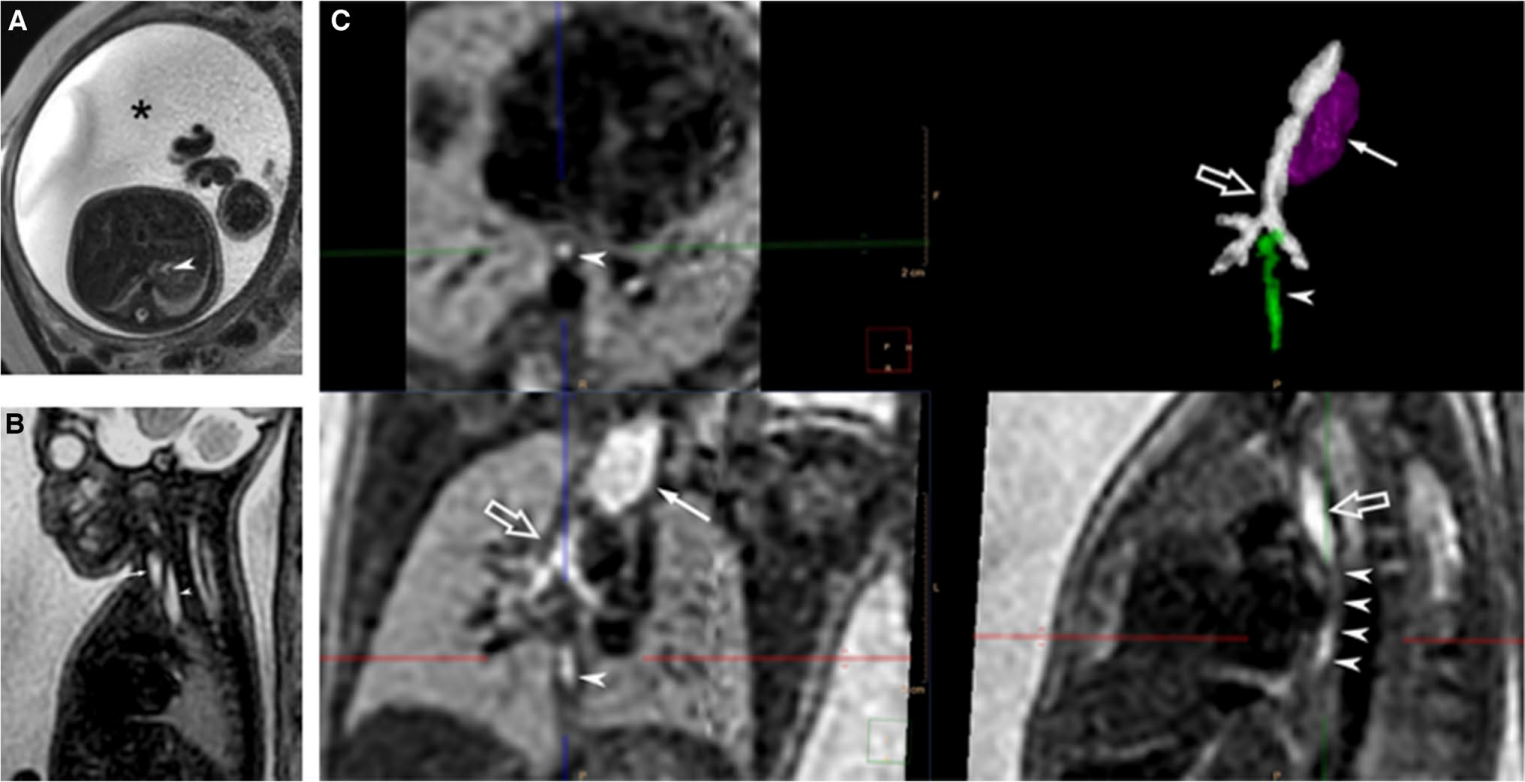
T2-weighted single-shot fast spin echo (SS-FSE) fetal MRI performed at 30 2/7 weeks using a 1.5-T scanner (Philips Achieva dStream, Philips Healthcare, Amsterdam, The Netherlands). **A** Axial image at the level of the upper abdomen shows polyhydramnios (*asterisk*) and small stomach (*arrowhead*). **B** Midline sagittal view of the chest shows a dilated upper esophageal pouch (*arrowhead*) posterior to the trachea (*arrow*). **C** Super-resolution SVR images of the fetal chest displayed in orthogonal axial, coronal, and sagittal planes. The images were manually segmented to produce the 3D-rendered image of the esophageal atresia with distal fistula. The atretic esophageal segment resulted in a dilated upper esophageal pouch (*arrows*) which was segmented out and colored *dark magenta*, the trachea and bronchi were segmented in *white* (*open arrows*), and the distal esophagus fistulized from the proximal left main stem bronchus (*arrowheads*) is colored *green*

Fetal ultrasound of a 27 2/7-week gestation male fetus showed a dilated right atrium, a single umbilical artery, a small fetal stomach, and a mild urinary tract dilation. A fetal MRI completed 3 weeks later showed polyhydramnios. The stomach was diminutive in size throughout the exam (Fig. 1A). A persistent dilated upper esophageal pouch with the distal esophagus contained a small amount of fluid (Fig. 1B). The images were post-processed using super-resolution imaging with SVR (Fig. 1C), showing esophageal atresia with a distal tracheoesophageal fistula arising from the left main stem bronchus. Additionally, the left kidney appeared to have a duplex collecting system or bifid renal pelvis and fetal heart examination showed a persistent left superior vena cava draining to the coronary sinus. Given the constellation of findings, there was concern for VACTERL association. Family history was unremarkable. The neonate was delivered at 30 4/7 weeks (birth weight = 1,590 g) and placed in the NICU. A chest and abdominal radiograph performed on DOL 0 showed that the distal tip of the nasogastric/orogastric tube projected over the upper esophagus. Tracheoesophageal fistula (TEF) was confirmed to arise from the left main stem bronchus adjacent to the carina and repaired on DOL 1 by rigid bronchoscopy and ligation of the TEF. Esophageal atresia repair was completed on DOL 4. Eight days later, the infant was extubated to CPAP. The patient remained hospitalized until DOL 106, with mainly complications of stage 2 bronchopulmonary dysplasia and bronchomalacia.

## Discussion

**Fig. 2:**
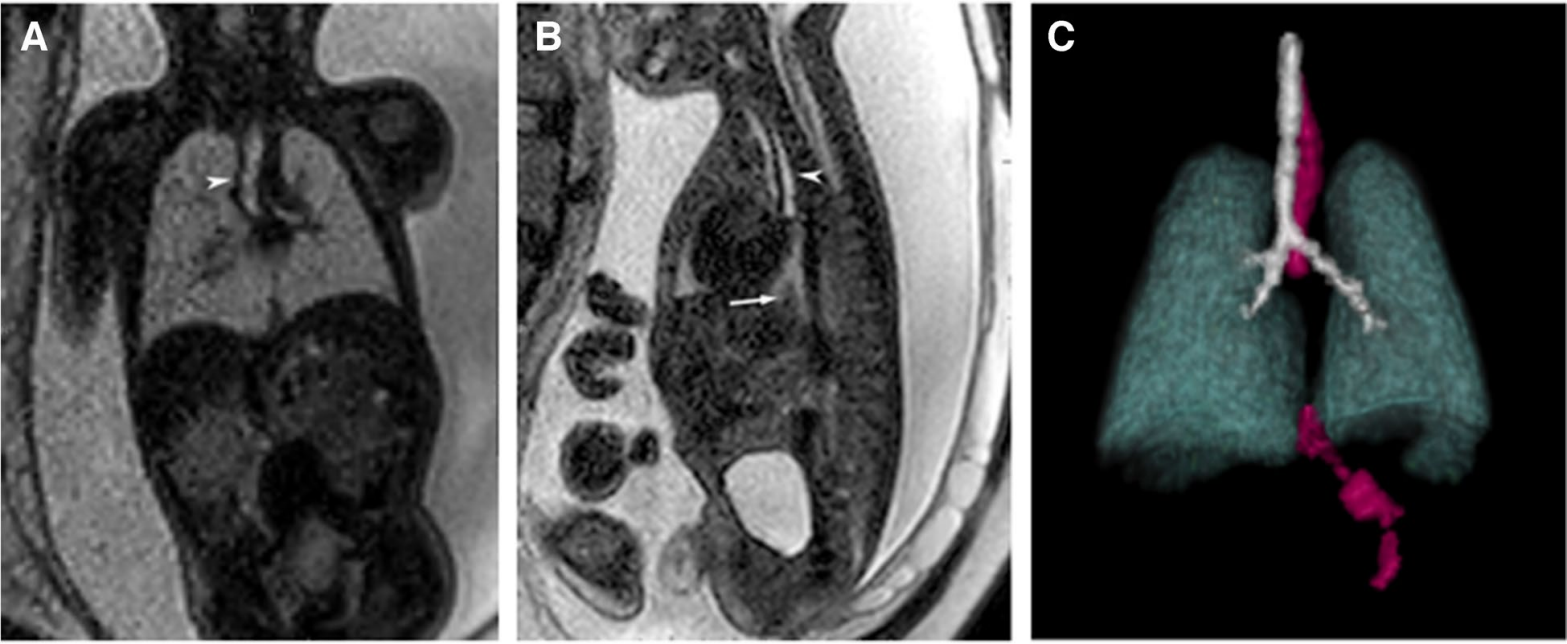
** A** Coronal and (**B**) sagittal T2-weighted SS-FSE fetal MRI images of a fetus with suspected esophageal atresia scanned at 33 2/7 weeks, showing a dilated upper esophageal pouch (*white arrowheads* on **A** and **B**). A large gap is seen between the atretic proximal esophagus and a small remnant of the distal esophagus (*white arrow* in **B**). **C** 3D-rendered images of the lungs (*green*), trachea and bronchi (*white*), and esophagus (*magenta*) show the atretic esophagus ending just below the carina and a large gap between the atretic upper esophagus and the distal gastroesophageal junction

The potential use of SVR to diagnose and classify esophageal atresia has been discussed with the idea that this technology may improve diagnostic accuracy and potentially identify a long-gap esophageal atresia which could guide surgeons in anticipating the need for esophageal replacement with a conduit [6]. To our knowledge, this is the first application of that idea. The case described in this report benefited from super-resolution SVR images and 3D rendering to prenatally detect an esophageal atresia with a distal esophageal fistula arising from the left main stem bronchus. A companion case examined at 33 2/7 weeks correctly diagnosed esophageal atresia without a distal fistula, demonstrating a gap between the esophageal pouch and the gastroesophageal junction (Fig. 2), which was confirmed during surgical repair on DOL 1.

This technique is novel in its use of SVR images and 3D rendering to assess and diagnose esophageal atresia and tracheoesophageal fistula structures. Although this approach is unlikely to impact the sensitivity and specificity of fetal MRI to diagnose esophageal atresia as the dilated upper esophageal pouch is identifiable in the original sequences [7], it has the potential to improve diagnostic precision by increasing the confidence to diagnose a fistula when present and, when not present, determine the presence and length of a gap. Identification and prediction of gap length may refine prenatal surgical counseling by identifying those cases that may require a conduit to reconstruct the esophagus, besides alerting the prospective parents for short- and long-term morbidity [8]. It may also allow for customization of tissue-engineered constructs to replace the esophagus in the future [6].

In conclusion, super-resolution SVR images and 3D rendering can be used to evaluate and visualize pre-surgical anatomy of neonatal esophageal atresia and tracheoesophageal fistula. The cases presented herein demonstrate the feasibility, but further studies are required to determine how consistently the technique can be used when applied to larger populations of fetuses at risk for esophageal atresia examined by fetal MRI.

## Data Availability

No datasets were generated or analysed during the current study.

## References

[1] Centini G, Rosignoli L, Kenanidis A, Petraglia F (2003) Prenatal diagnosis of esophageal atresia with the pouch sign. Ultrasound Obstet Gynecol 21:494–497 12768564 10.1002/uog.58

[2] Arntzen T, Mikkelsen A, Emblem R, Lai X, Haugen G (2023) Prenatal diagnosis of esophageal atresia – performance and consequences. J Ped Surg 58(11):2075–2080 10.1016/j.jpedsurg.2023.05.015 37407414

[3] Pardy C, D’Antonio F, Khalil A, Giuliani S (2019) Prenatal detection of esophageal atresia: a systematic review and meta-analysis. Acta Obstet Gynecol Scand 98(6):689–699. 10.1111/aogs.13536 30659586 10.1111/aogs.13536

[4] Coakley FV, Glenn OA, Qayyum A, Barkovich AJ, Goldstein R, Filly RA (2004) Fetal MRI: a developing technique for the developing patient. AJR Am J Roentgenol 182(1):243–252. 10.2214/ajr.182.1.1820243 14684546 10.2214/ajr.182.1.1820243

[5] Uus A, Zhang T, Jackson LH, Roberts TA, Rutherford MA, Hajnal JV (2020) Deformable slice-to-volume registration for motion correction of fetal body and placenta MRI. IEEE Trans Med Imaging 39:2750–2759. 10.1109/TMI.2020.2974844.PMID32086200 32086200 10.1109/TMI.2020.2974844 PMC7116020

[6] Davidson JR, Uus A, Matthew J, Egloff AM, Deprez M, Yardley I, De Coppi P, David A, Carmichael J, Rutherford MA (2021) Fetal body MRI and its application to fetal and neonatal treatment: an illustrative review. Lancet Child Adolesc Health 5(6):447–458. 10.1016/S2352-4642(20)30313-8 33721554 10.1016/S2352-4642(20)30313-8 PMC7614154

[7] Hochart V, Verpillat P, Langlois C, Garabedian C, Bigot J, Debarge VH, Sfeir R, Avni FE (2015) The contribution of fetal MR imaging to the assessment of oesophageal atresia. Eur Radiol 25(2):306–314. 10.1007/s00330-014-3444-y 25304819 10.1007/s00330-014-3444-y

[8] Koivusalo A, Pakarinen MP, Turunen P, Saarikoski H, Lindahl H, Rintala RJ (2005) Health-related quality of life in adult patients with esophageal atresia–a questionnaire study. J Pediatr Surg 40(2):307–312. 10.1016/j.jpedsurg.2004.10.014 15750920 10.1016/j.jpedsurg.2004.10.014

